# A facile preparation method for new two-component supramolecular hydrogels and their performances in adsorption, catalysis, and stimuli-response

**DOI:** 10.1039/c9ra03827b

**Published:** 2019-07-22

**Authors:** Junlin Zhu, Ran Wang, Rui Geng, Xuan Zhang, Fan Wang, Tifeng Jiao, Jingyue Yang, Zhenhua Bai, Qiuming Peng

**Affiliations:** State Key Laboratory of Metastable Materials Science and Technology, Yanshan University Qinhuangdao 066004 P. R. China tfjiao@ysu.edu.cn; Hebei Key Laboratory of Applied Chemistry, School of Environmental and Chemical Engineering, Yanshan University Qinhuangdao 066004 P. R. China yangjingyue@ysu.edu.cn; National Engineering Research Center for Equipment and Technology of Cold Strip Rolling, Yanshan University Qinhuangdao 066004 P. R. China

## Abstract

In this study, we prepared a novel multifunctional two-component supramolecular hydrogel (T-G hydrogel) *via* two organic molecules in ethanol/water mixed solvents. In addition, we prepared gold nanoparticle/T-G (AuNPs/T-G) composite hydrogels using T-G hydrogel as a template for stabilizing AuNPs by adding HAuCl_4_ and NaBH_4_ during the heating and cooling process of T-G hydrogels. The morphology and microstructure of the as-prepared hydrogels were characterized using SEM, TEM, XRD, and FT-IR. The hydrogels prepared by solutions that contained different ethanol/water volume ratios exhibited different microstructures, such as sheets, strips, and rods. The obtained T-G hydrogels exhibited a sensitive response to pH changes in the process of sol–gel transformation and showed good adsorption properties for model organic dyes. In the presence of NaBH_4_, the obtained AuNP/T-G composite hydrogels exhibited the excellent catalytic performance for 4-nitrophenol (4-NP) degradation. Thus, the current research provides new clues in developing new multifunctional two-component supramolecular gel materials and exhibits potential applications for wastewater treatment.

## Abstract 2

New two-component supramolecular hydrogels were prepared *via* a self-assembly process, demonstrating potential applications in adsorption and catalysis as well as sensor materials.

## Graphical abstract

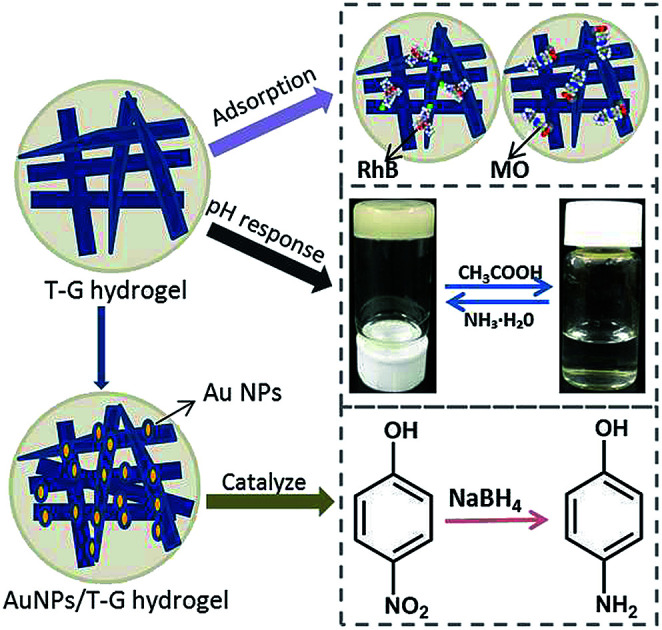

## 1 Introduction

Supramolecular gels are commonly formed by the self-assembly of low molecular weight organic compounds through interactions, such as intermolecular non-covalent bonding van der Waals forces, electrostatic interactions, and hydrogen bonding, forming a one-dimensional fiber or ribbon-like structure.^1,2^ Then, the formed one-dimensional structure intertwines, to form a three-dimensional network structure, thereby allowing the solvent to solidify and form gels.^3^ Unlike polymer gels, supramolecular gel fibers are connected by non-covalent bonds, due to which they show a certain response to external stimuli, such as temperature, pH, light, ultrasound, chemicals, and electromagnetic fields.^4–6^ In addition, supramolecular gels have plasticity, and the properties of supramolecular gels can be changed by modifying the gel factor preparation process and structure. Therefore, supramolecular gels have attracted increasing attention in recent years.^7,8^ So far, the small molecule organogels, such as amino acids, hydrocarbons, amides, and cholesterol have been reported.^9–14^ Recently, two-component supramolecular hydrogels have been widely used due to their numerous functionalized groups and tightly connected three-dimensional network structures.^15–21^ For example, Takeshita *et al.* constructed an amphiphilic tris-urea-based supramolecular hydrogel *via* the self-assembly of small molecules and studied its adsorption properties for the organic dyes.^22^ Zhang *et al.* threaded β-cyclodextrin onto PPG-NH_2_ chains and the resultant pseudorotaxanes interacted with the CNS matrix to construct supramolecular hydrogels, which presented good adsorption properties for the cationic dyes.^23^ Qu *et al.* constructed a peroxidase/oxidase enzyme system based on a supramolecular hydrogel and investigated its application on cascade reactions as a biocatalyst.^24^ Potier *et al.* showed that the addition of methylated β-cyclodextrin in the supramolecular hydrogel could exert a positive effect on the catalytic activity of the hydroformylation reaction.^25^ Guan *et al.* reported the formation of a novel hydrogel *via* a host–guest complexation between PNIPAM and a cyclodextrin dimer, and the hydrogel responded sensitively to temperature, light, and reduction stimuli, in the form of a sol–gel conversion.^26^

**Fig. 1:**
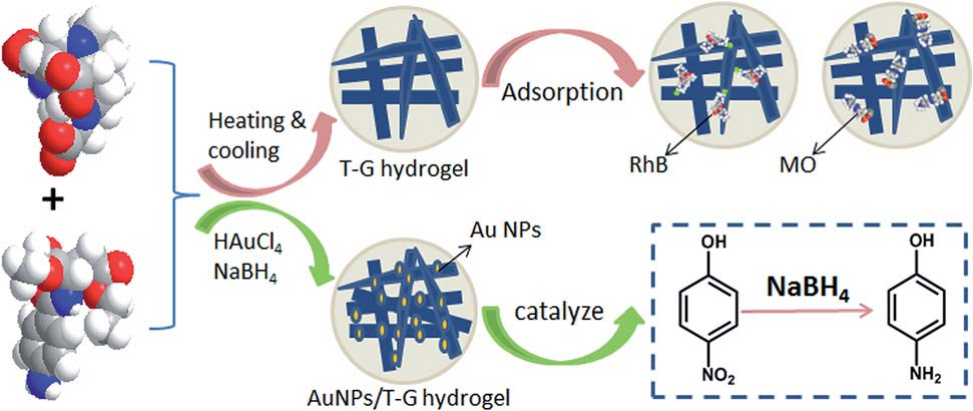
Schematic of adsorption of the T-G hydrogel and the catalysis of the AuNP/T-G hydrogel.

In this work, we studied a self-assembled multifunctional two-component supramolecular hydrogel based on two kinds of organic small molecules, namely, 1,4,7,10-tetraazacyclododecane-1,4,7-triacetic acid and *N*-(4-aminobenzoyl)-l-glutamic acid diethyl ester that underwent self-assembly *via* inter-molecular non-covalent bonding to form a one-dimensional fiber or band structure. Then, the one-dimensional structures interweaved together to form a three-dimensional network structure. The obtained hydrogel responded to pH changes in the form of a sol–gel transformation and had good adsorption capacities for the organic dyes. As shown in Fig. 1, the composite gel prepared by using the hydrogel as a template to stabilize gold nanoparticles showed good catalytic degradation performance for 4-NP. To the best of our knowledge, this is a novel multifunctional glutamic acid derivative-based supramolecular hydrogel with adsorption, simulation response, and catalytic properties. The obtained hydrogels can be used widely in the fields of wastewater treatment, dye adsorption, and drug release.

## 2 Materials and methods

### 2.1 Materials

*N*-(4-Aminobenzoyl)-l-glutamic acid diethyl ester (99%), chloroauric acid (HAuCl_4_·4H_2_O), NaBH_4_, and 4-nitrophenol (4-NP) were purchased from Alfa Aesar (Tianjin, China) Chemicals. 1,4,7,10-Tetraazacyclododecane-1,4,7-triacetic acid (98%) was purchased from Sigma-Aldrich (Shanghai, China) and used without further purification. The used Rhodamine B (RhB) and methyl orange (MO) dyes were purchased from Beijing Chemicals. Acetic acid (analytically pure, CH_3_COOH, % > 99.5) and ammonia water (NH_3_, wt% = 23–25) were purchased from Aladdin Reagent Chemicals (Shanghai, China). All ultrapure water was purchased from a Millipore Milli-Q purification system.

### 2.2 Preparation of the T-G hydrogel

There exists a convenient method to prepare current supramolecular two-component hydrogels. In this method, 1,4,7,10-tetraazacyclododecane-1,4,7-triacetic acid (11.5 mg) and *N*-(4-aminobenzoyl)-l-glutamic acid diethyl ester (32.3 mg) were first mixed in the molar ratio of 1 : 3. Then, the mixed solvents (1 mL) with different volume ratios of ethanol/water were added to glass bottles, and the solution was sonicated for 5 minutes to obtain a good dispersion. The ultrasonic power of sonicator was 360 W with a frequency of 40 kHz. Subsequently, the solution was heated for 20 minutes in a water bath at 80 °C. Then, the solution was allowed to naturally cool to room temperature, and the glass bottle was inverted to observe whether the gel was formed.

### 2.3 Preparation of the AuNP/T-G hydrogel

First, 1,4,7,10-tetraazacyclododecane-1,4,7-triacetic acid (11.5 mg) and *N*-(4-aminobenzoyl)-l-glutamic acid diethyl ester (32.3 mg) were mixed in the molar ratio of 1 : 3. Then, 0.33 mL ethanol, 0.12 mL ultra-pure water, and 0.33 mL HAuCl_4_·4H_2_O (10 mmol L^−1^) were added into the bottle, and the solution was sonicated for 5 minutes to obtain a good dispersion. Subsequently, the solution was heated for 20 minutes in a water bath at 80 °C. Then, 0.22 mL of NaBH_4_ (1 mg mL^−1^), dissolved in a 0.3 mol L^−1^ NaOH solution, was added into the bottle. Then, the solution was allowed to naturally cool to room temperature to form the hydrogel. Eventually, the formed AuNP/T-G hydrogel was subjected to catalytic tests.

### 2.4 Adsorption experiment tests

The adsorption tests were performed at room temperature. Approximately, 1 mL of the T-G hydrogel was added to the dye solution (RhB, 12 mg L^−1^, 50 mL; MO, 6 mg L^−1^, 50 mL). The dye solution was then magnetically stirred at room temperature. Then, the supernatant of the dye solution was added to a quartz cuvette for subsequent testing at different time intervals. The absorbance was measured at a wavelength of 554 nm (RhB) and 463 nm (MO) using an UV-vis spectrophotometer.

### 2.5 Catalytic experiment tests

2.8 mL of 4-NP (0.1 mmol L^−1^), 0.2 mL of NaBH_4_ (0.1 mol L^−1^), and 100 mg of the AuNP/T-G hydrogel were mixed in a quartz cuvette. Then, the spectrum in the wavelength range of 200–800 nm was measured using an UV-vis spectrophotometer at certain time intervals, and the scanning was continued until the absorbance was close to a straight line at 401 nm. By this time, it was found that the yellow color of 4-NP disappeared.

### 2.6 Characterization

For the characterization experiments, the solvents were first removed from the as-prepared gels by drying them on an FD-1C-50 lyophilizer from Beijing Boyikang Experimental Instrument Company (Beijing, China). For the SEM measurement, the lyophilized samples coated on a copper foil were sprayed with Au, and then fixed on the sample stage using a conductive tape. The instrument used was Hitachi S-4800 field emission scanning electron microscope, whose acceleration voltage was 5–15 kV. The TEM images of different lyophilized samples were recorded on a transmission electron microscope (HT7700, Hitachi High-Technologies Corporation, Japan). The FT-IR spectra of different lyophilized samples were recorded on a Nicolet spectrophotometer of Thermo Fisher Scientific Inc. The UV-Vis spectra were recorded on an UV-TU1810PC spectrophotometer. The XRD patterns were recorded on a Rigaku D/max 2550 PC diffractometer (Rigaku Inc., Tokyo, Japan) using Cu Kα radiation at 40 kV voltage and 200 mA current. The specific surface area and the pore size distribution of the as-prepared hydrogels were measured using a Brunauer–Emmett–Teller measurement (NOVA 4200-P). The Dynamic rheology test was measured on an Anton Paar MCR302 rheometer at room temperature.

## 3 Results and discussion

### 3.1 Gelation behaviors and characterizations

**Fig. 2:**
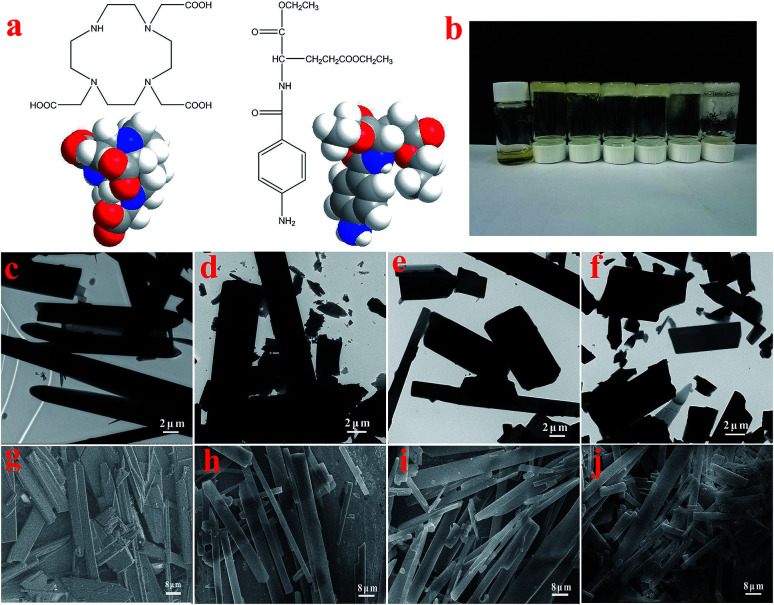
Molecular structures and 3D space-filling models of T-G (a), picture of T-G hydrogels with different ethanol/water ratios (b), TEM images of the lyophilized samples with ethanol/water ratios of 1 : 1, 1 : 2, 1 : 3, and 1 : 4 (c–f), SEM images of the lyophilized samples with ethanol/water ratios of 1 : 1, 1 : 2, 1 : 3, and 1 : 4, respectively (g–j).

The molecular structures and the 3D model of 1,4,7,10-tetraaza-cyclododecane-1,4,7-triacetic acid and *N*-(4-aminobenzoyl)-l-glutamic acid diethyl ester (T-G) are shown in Fig. 2a. Table 1 demonstrates the gelation behaviors of the two-component mixture in different solvents. The results show that the two-component mixture could form a non-transparent supramolecular gel in different solvents. Particularly, when the ethanol/water ratio changed from 1 : 1 to 1 : 4, hydrogels (abbreviated as Gel-11, Gel-12, Gel-13, and Gel-14) were formed in the system. At this point, we use S, PS, PG, and G to represent the solution, part solution, part gel, and gel states, respectively. We define *Q* as the ratio of gel volume to the total system volume. Specifically, when 0 < *Q* < 0.1, the system is in a solution state; 0.11 < *Q* < 0.5, the system is in a part solution state; 0.51 < *Q* < 0.9, the system is in a part gel state; 0.91 < *Q* < 1.0, the system is in a gel state. However, when the ethanol/water ratio was more than 1 : 1 or less than 1 : 4, the mixtures were found to be in the S, PG, or PS states. It could be considered that dipole interactions and the hydrogen bonding between the solvent and the gel factors were the main reasons for influencing the stability of gels. In general, the hydrogen bonding of a solvent affects the properties of supramolecular hydrogels more significantly than any other intermolecular forces. The stronger the hydrogen bonding ability of the solvent the easier it is to form the hydrogen bonds with the gel factor, and worse is the stability of the gel.^27^

**Table 1:** Gelation behaviors of T-G with different solvent ratios^a^

Ethanol/water	T-G
2 : 1	S
1 : 1	G
1 : 2	G
1 : 3	G
1 : 4	G
1 : 5	PG
1 : 10	PS

a S: solution; PS: part solution; PG: part gel; G: gel.

As shown in Fig. 2, for the sake of understanding the microstructure of the hydrogel prepared in this experiment, it was analyzed using TEM and SEM. In Fig. 2c–f, it can be observed that the microstructures of the lyophilized samples with different solvents were in the form of sheets, strips or rods with the length of several tens of microns, thereby demonstrating the microstructure of a typical two-component organic molecular physical gel.^28–30^ The size and degree of chaos of the sheets varied with the volume ratio of the mixed solvents. Among them, the sheets of Gel-12 have the largest size with the most uniform distribution. Further, the SEM images in Fig. 2g–j clearly show the microscopic morphologies of different hydrogels, showing a corresponding structure to the TEM images. The different gel morphologies may be related to solubility, solvent hydrogen bonding, and spatial steric hindrances, which have an important impact on the gel formation process.

**Fig. 3:**
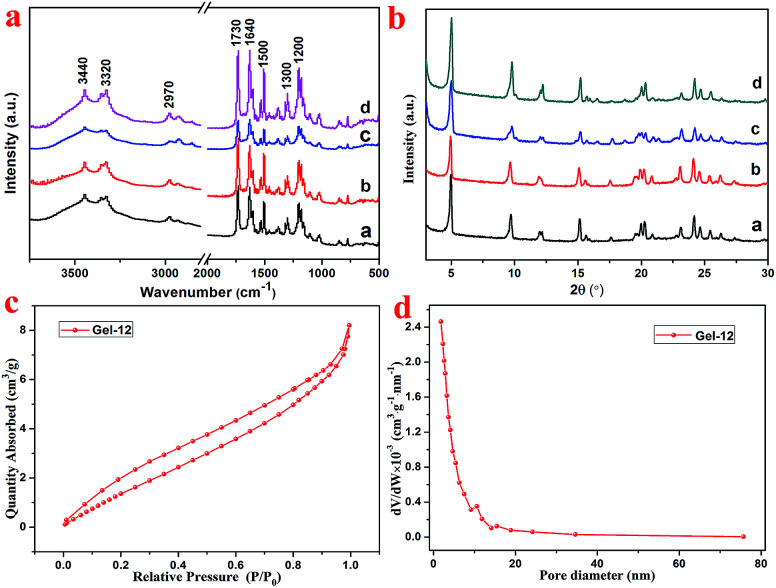
The FT-IR spectra of the lyophilized samples of the T-G hydrogel (a, b, c, and d corresponding to ethanol/water ratios of 1 : 1, 1 : 2, 1 : 3, and 1 : 4) (a), X-ray diffraction patterns of the lyophilized samples of the T-G hydrogel (a, b, c, and d corresponding to ethanol/water ratios of 1 : 1, 1 : 2, 1 : 3, and 1 : 4) (b), nitrogen adsorption–desorption isotherms of Gel-12 (c), and pore size distributions of Gel-12 (d).

As shown in Fig. 3a, the FT-IR spectra of the lyophilized samples with different solvents were analyzed to further study the mechanism of self-assembly. It is clear that significant characteristic peaks observed at 3440, 3320, 2970, 1730, 1640, 1500, 1300, and 1200 cm^−1^, correspond to N–H stretching, methylene stretching, C=O ester stretching, amide I band, and benzene ring stretching. This indicates that there are characteristic functional groups and intermolecular hydrogen bonding in the as-prepared hydrogels. Moreover, as shown in Fig. 3b, for the sake of exploring the well-organized accumulation and solvent effect in the microstructure of gels,^31^ the XRD curves of the lyophilized samples of T-G hydrogels with different solvents were tested. The curves of the lyophilized samples exhibit obvious peaks at 2*θ* values of 4.97°, 9.71°, 15.15°, 17.64°, 20.26,° and 24.11°, with the d values corresponding to 2*θ* values of 1.8, 0.91, 0.58, 0.50, 0.44, and 0.37 nm. The significant changes observed in the spectra of the lyophilized samples with different solvents demonstrated the existence of different hydrogen-bond interactions in the as-prepared hydrogels. It can be noticed that as the volume ratio decreases, the peak intensity at 4.97° first decreases and then increases, while the peak intensity at 9.71° exhibited a similar trend. The results of this experiment reveal that the volume ratio of ethanol/water has an important influence on the self-assembly process of the supramolecular gels. As shown in Fig. 3c and d, the porous microstructures of the hydrogels were studied using the nitrogen adsorption–desorption isotherms, and the pore size distribution of the selected hydrogel was evaluated using the BJH method. Gel-12 was selected as the representative target. Within the tested pressure range, Gel-12 showed a hysteresis loop of the IV isotherm curve at *p*/*p*_0_ = 0.1–0.9, representing the mesoporous structure in Gel-12.^32^ The BJH method was also used to evaluate the physical properties of the Gel-12. The experimental data show that the specific surface area of Gel-12 is 9.837 m^2^ g^−1^, while the average pore diameter is 5.197 nm and the average pore volume is 0.012781 cm^3^ g^−1^.

**Fig. 4:**
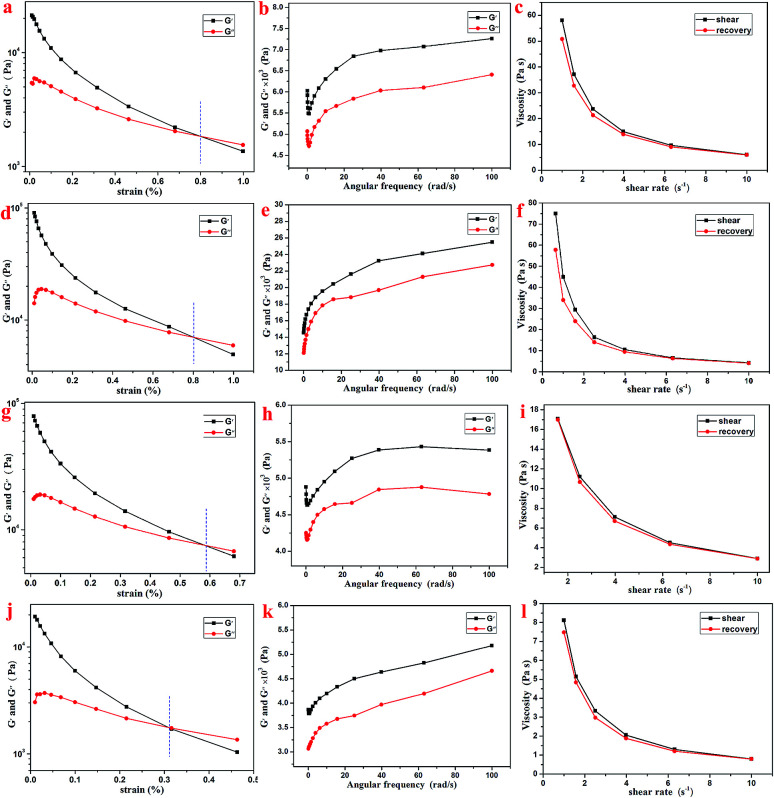
Rheological characterizations of Gel-11 (a–c), Gel-12 (d–f), Gel-13 (g–i) and Gel-14 (j–l).

Further, the rheology performance of the T-G hydrogels was studied. As we know, the storage modulus (*G*′) indicates the amount of energy stored when the material is elastically deformed, while the loss modulus (*G*′′) indicates the amount of energy lost when the material is viscously deformed. Upon comparing Fig. 4a, d, g, and j, it is observed that the *G*′ and *G*′′ values of the Gel-11, 12, 13, and 14 were equal at shear stresses of 0.79, 0.81, 0.58, and 0.31%, respectively. As shown in Fig. 4b, e, h, and k, the oscillating shear rheological behavior of the as-prepared gels were tested when the shear stress was 0.001%. The experimental results showed that *G*′ was dominant in the tested frequency range, thereby showing a true gel state.^33^ Further, the curves in Fig. 4c, f, i, and l indicate that the as-prepared gels can be substantially shear-thinned, so it could be concluded that they have good self-healing properties. Thus, the results mentioned above reveal that the viscoelastic region and the shear strength of Gel-12 were maximum in all the gels, and the one-dimensional structure of Gel-12 bonds more tightly than other gels. In other words, Gel-12 was tougher than the other three gels, macroscopically. This means that Gel-12 is more difficult to collapse in the solution, which is advantageous for its subsequent applications.

### 3.2 Absorption capacity of the T-G hydrogel

**Fig. 5:**
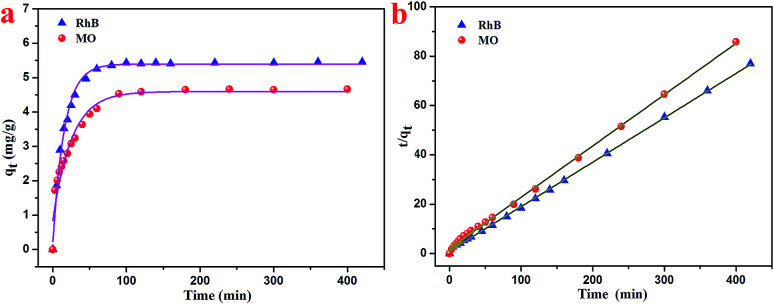
Adsorption kinetics curves of the as-prepared T-G hydrogels on RhB and MO at 298 K.

Based on the characterizations, it was found that the as-prepared hydrogel had a porous microstructure and a large specific surface area. Therefore, the adsorption performance of the currently prepared hydrogel was investigated. Gel-12 was chosen as the test adsorbent for the selective removal of RhB and MO because of the tight binding of its one-dimensional structures. It was difficult for Gel-12 to collapse in the solution, which proved to be advantageous for the adsorption experiments. As shown in Fig. 5, the as-prepared hydrogel exhibited a continuous adsorption for the two dyes, with the equilibrium time of RhB being about 100 minutes, whereas for MO, it was about 180 minutes. Thus, the porous microstructures of the as-prepared hydrogel played an important role in the adsorption process through many weak interactions.^34–36^ Therefore, the classical kinetic models were utilized to show the above adsorption mechanism in the following manner:

The pseudo-first-order model:1
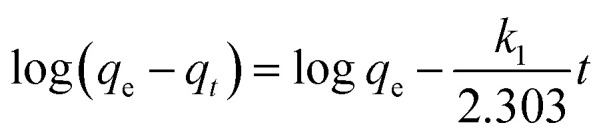


The pseudo-second-order model:2
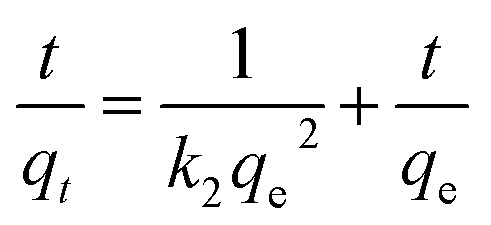
where *q*_e_ represents the amount of the adsorbed dye at equilibrium time, *q*_*t*_ represents the amount of the adsorbed dye at time *t* (mg g^−1^), and *k*_1_ and *k*_2_ represent the kinetic rate constants. The adsorption test results are summarized in Table 2. The adsorption results show that the pseudo-first-order model has a higher correlation coefficient (*R*^2^ > 0.99) in both the RhB and MO adsorption processes, which was more accurate than the pseudo-second-order model. Furthermore, the dye adsorption efficiency of Gel-12 for RhB was 5.4 mg g^−1^, and for MO, it was 4.6 mg g^−1^. In view of the adsorption experimental data, the supramolecular gel prepared in this experiment showed a better adsorption efficiency for the two dyes using the pseudo-first-order-model than the pseudo-second-order model. In addition, compared with various hydrogels listed in Table 3, the present hydrogels showed acceptable capacities towards common dyes in the wastewater treatment.

**Table 2:** The corresponding adsorption model parameters of the as-prepared hydrogel

Dye	Pseudo-first-order model			Pseudo-second-order model		
	*q*_e_ (mg g^−1^)	*R* ^2^	*k*_1_ (min^−1^)	*q*_e_ (mg g^−1^)	*R* ^2^	*k*_2_ (g mg^−1^ min^−1^)
RhB	5.40	0.9922	0.064	5.56	0.9995	0.031
MO	4.60	0.9483	0.039	4.81	0.9988	0.022

**Table 3:** The absorption capacities of other reported hydrogels regarding RhB and MO

	*q*_e_ (mg g^−1^)	Reference
**RhB**		
MOG-1	4.80	37
PAA-Ag/AgNP hydrogel	3.41	38
RGO-CS	1.83	39
T-G hydrogel	5.40	This study

**MO**		
HNTs	2.0	40
FC hybrid gel	2.2	41
CD-0	19	42
T-G hydrogel	4.6	This study

### 3.3 Sol–gel transformation of the T-G hydrogel

Supramolecular gel factors are simple to synthesize and easy to modify, and various functional groups can be introduced into the hydrogel, which allows the supramolecular gels to initiate response phenomena under environmental stimuli, such as ultrasound, pH, ions, mechanical force, and electromagnetic field. The response phenomena include gel color change, gel–sol phase transition, gel–gel phase transition, isomerization, the formation of dimers, and morphological changes. The sensitivity of the supramolecular gels towards the environmental stimuli makes them suitable for application in materials science,^43^ bio-polymers,^44^ photo-switch,^45^ and many other fields.

**Fig. 6:**
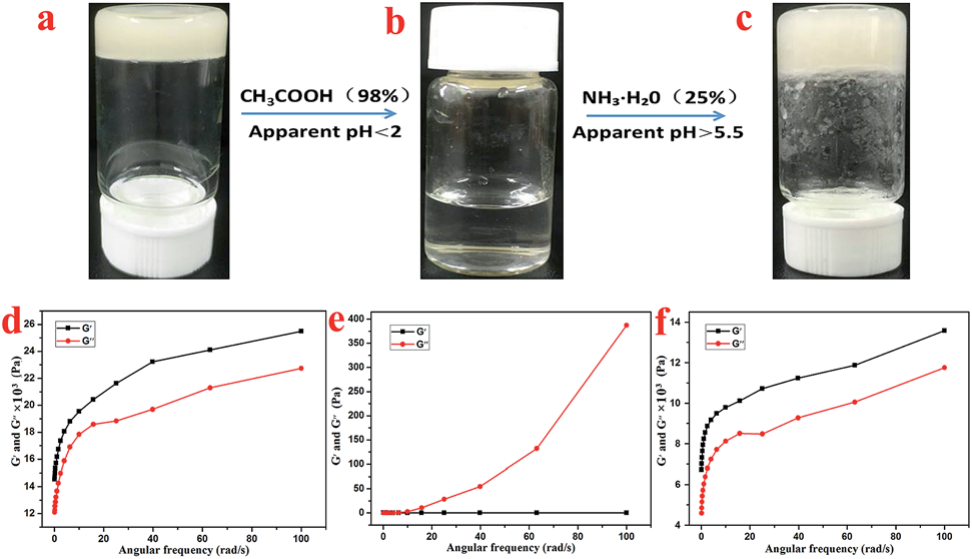
The sol–gel transformation of the T-G hydrogel in response to alteration of acidity at room temperature (a–c), and the corresponding rheological characterizations (d–f), respectively.

As shown in Fig. 6a–c, the hydrogel with 1 mL volume was inverted in a small glass bottle without flowing, and the pH of the initial state hydrogel was 3.0. Then, 0.35 mL of acetic acid was added to the hydrogel system, and the hydrogel completely dissolved within 1 minute with the final pH of the solution being 2.0. Then, through subsequent addition of 0.5 mL of ammonia water into the small glass bottle, an opaque hydrogel was formed once again, but the pH of the hydrogel was 5.5. This process could also be characterized by rheological testing. As shown in Fig. 6a and d, the system is in a gel state at this time, and it is found that *G*′ dominates the detected frequency range and exhibits a true gel state behavior. After the addition of acetic acid, the gel transformed into a liquid, as shown in Fig. 6b and e. At this time, *G*′ is less than *G*′′ in the detected frequency range, thereby exhibiting a liquid behavior. However, after the addition of ammonia water, the system reverted back to the gel state, as shown in Fig. 6c and f. At this time, *G*′ dominates the detected frequency range again, thus exhibiting a gel state behavior. Therefore, it can be concluded that the T-G hydrogel exhibits a sensitive response to pH changes.

### 3.4 Preparation and catalytic performance of the AuNP/T-G hydrogel

The surface effect of the metal nanoparticles has broad application prospects in organic catalysis. However, the metal nanoparticles easily agglomerate, which reduces their catalytic activity. Therefore, it is of great practical significance to solve the agglomeration problem of the metal nanoparticles. A supramolecular hydrogel having a three-dimensional network structure and a large number of functional groups can provide space for the nucleation and growth of the noble metal nanoparticles, and thus a supramolecular hydrogel can be used as a template or support for the synthesis of the precious metal nanoparticles.^46,47^

**Fig. 7:**
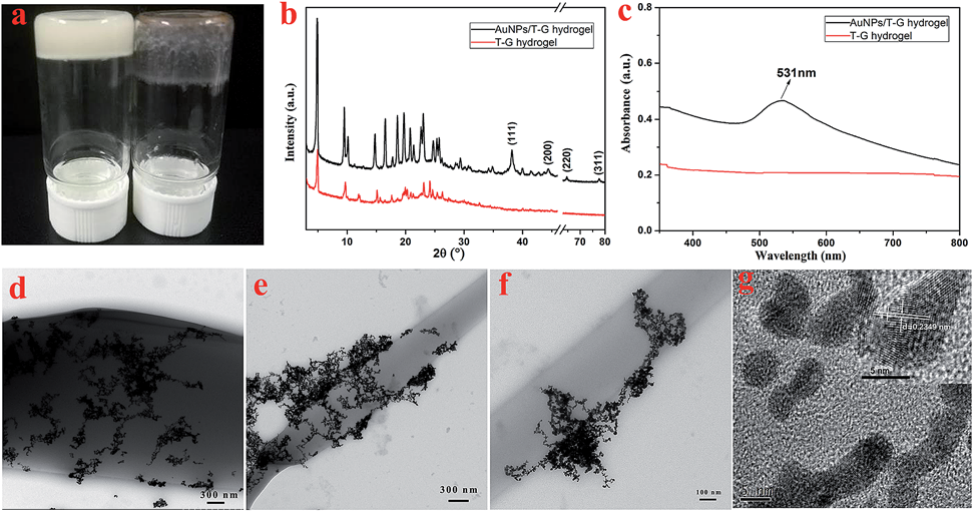
The photograph of the T-G hydrogel and AuNP/T-G hydrogel (a), XRD pattern (b) and UV-vis absorption spectra (c) of the T-G hydrogel and AuNP/T-G hydrogel, TEM images of the AuNP/T-G hydrogel (d–f), and HRTEM of AuNPs (g).

*N*-(4-Aminobenzoyl)-l-glutamic acid diethyl ester, in this case, carries a number of protonated –NH_2_ groups, which have electrostatic interactions with AuCl_4_^−^. It is worth noting that the as-prepared Au nanoparticles exhibit good dispersibility and are likely to exhibit good catalytic properties. As shown in Fig. 7b, the XRD pattern of the obtained AuNP/T-G hydrogel demonstrated the newly generated diffraction peaks of 2*θ* values at 38.1°, 44.3°, 64.5°, and 77.6°, which corresponded to the (111), (200), (220), and (311) face-centered cubic structures of the gold nanoparticles. Fig. 7c exhibits the UV-visible absorption spectrum of the AuNP/T-G hydrogels with AuCl_4_^−^ concentration of 10 mmol L^−1^. Moreover, there is an obvious peak at 531 nm, which corresponds to the characteristic plasmon resonance band of Au NPs, suggesting the formation of AuNP. Further, TEM was performed to study the obtained AuNP/T-G hydrogels (Fig. 7d–f). The gold nanoparticles adhere to the surface of the one-dimensional rods of the hydrogel without significant aggregation. The above results demonstrated that the T-G hydrogel provided a template for the attachment of AuNPs. As shown in Fig. 7g, the interplanar spacing of Au nanoparticles is 0.2349 nm, which matches well with the (111) crystal surface of Au.

**Fig. 8:**
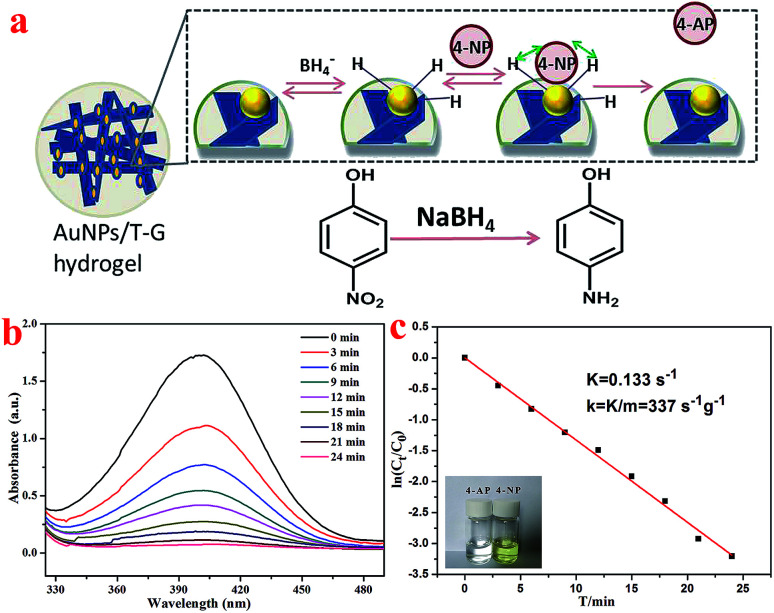
Schematic of the Langmuir–Hinshelwood model for the catalytic reduction of 4-NP (a), UV-vis spectra for the catalytic reduction of 4-NP (b), plots of ln(*C*_*t*_/*C*_0_) *versus T* for reduction process with the inserted photo of color change from 4-NP to 4-AP (c).

4-NP has become a harmful water pollutant because of its nitro group, which has aroused the attention of people from all over the world.^48^ However, 4-AP containing a –NH_2_ group is widely used in a lot of industries, including pharmaceuticals and dye intermediates.^49–51^ The process of the catalytic reduction of 4-NP to 4-AP can be detected using the UV-vis absorption spectroscopy because a continuous decrease in the absorption peak at 401 nm can be observed during the reaction. As shown in Fig. 8a, in the presence of the as-prepared Au nanoparticles as a nanocatalyst, the mechanism of reducing 4-NP to 4-AP by NaBH_4_ conforms to the Langmuir–Hinshelwood model.^52,53^Fig. 8b shows the UV-visible absorption spectra of the 4-NP and NaBH_4_ solution under the catalysis of gold nanoparticles. The experimental results revealed that as the reaction time increases, the absorption peak at 401 nm gradually decreases until it disappears completely. This conversion process was also proven by the color change of the solution.

In the catalytic reaction of 4-NP, the concentration of NaBH_4_ was considered to be a certain value because it was two orders of magnitude higher than the concentration of 4-NP. For the above reason, the pseudo-first-order kinetic was chosen to estimate the rate constant (*K*) of the catalytic reaction of 4-NP. Fig. 8c shows the relationship between ln(*C*_*t*_/*C*_0_) and reaction time (*T*) during the catalytic reduction reaction. It could be concluded that the catalytic reduction reaction conforms to the pseudo-first-order kinetic model because a linear relationship exists between ln(*C*_*t*_/*C*_0_) and *T*. The *K* value was determined to be 0.133 s^−1^ based on the slope of the fitted curve. Consequently, the value of the kinetic activity coefficient *k* was calculated to be 337 g^−1^ s^−1^ (*k* = *K*/*m*, *m* represents the mass of the catalyst added). Thus, present obtained composite hydrogels demonstrated a new clue for the design of self-assembled nanomaterials and organized composites.^54–65^

## 4 Conclusions

In summary, new multifunctional supramolecular two-component T-G hydrogels were prepared, and their sol–gel transformation and adsorption performance were studied. In addition, the porous microstructure of the T-G hydrogels demonstrated a large specific surface area, which favored the anchoring and dispersion of the following loaded gold nanoparticles. Further, the obtained results showed that the T-G hydrogels demonstrated a sensitive response to pH changes and good adsorption properties for organic dyes. Therefore, it could be concluded that the T-G hydrogels showed good adsorption capacity for RhB and MO, with pseudo-first-order model fitting more appropriately than the pseudo-second-order. In addition, the AuNP/T-G composite hydrogels showed excellent catalytic activity for 4-NP. Thus, this study provided a new clue for the preparation of novel multifunctional two-component supramolecular gels and their potential applications for wastewater treatment. However, there are still some aspects to be addressed in the complicated process of wastewater treatment.

## Conflicts of interest

There are no conflicts to declare.

## Supplementary Material
